# Proposal of a new slit-lamp shield for ophthalmic examination and assessment of its effectiveness using computational simulations

**DOI:** 10.5935/0004-2749.20230058

**Published:** 2023

**Authors:** Daniel Araújo Ferraz, Zeyu Guan, Edinilson A. Costa, Eduardo Martins, Pearse A. Keane, Daniel Shu Wei Ting, Rubens Belfort Jr, Rafael Scherer, Victor Koh, Cristina Muccioli

**Affiliations:** 1 Department of Ophthalmology, Universidade Federal de São Paulo, São Paulo, SP, Brazil.; 2 NIHR Biomedical Research Centre for Ophthalmology, Moorfields Eye Hospital NHS Foundation Trust and UCL Institute of Ophthalmology, London, United Kingdom.; 3 Department of Mechanical Engineering, Universidade de São Paulo, São Paulo, SP, Brazil.; 4 Department of Biomedical Engineering Yonsei University, South Korea.; 5 Singapore Eye Research Institute, Singapore National Eye Centre, Duke-NUS Medical School, National University of Singapore, Singapore.; 6 Department of Ophthalmology, Universidade de São Paulo, São Paulo, SP, Brazil.; 7 Department of Ophthalmology, National University Hospital, Singapore.

**Keywords:** Ophthalmolog**i**sts, Coronavirus infections/prevention & control;Pandemics, Lipid droplets, SARS-CoV-2, Slit-lamp, Computer simulation, Protectivedevices, Equipment design, Oftalmologistas, Infeções por coronavírus/prevenção & controle, Pandemias, Gotículas lipídicas, SARS-CoV-2, Lâmpada de fenda, Simulação por computador, Equipamentos de proteção, Desenho de equipamento

## Abstract

**Purpose:**

This study aimed to use computational models for simulating the movement of respiratory droplets when assessing the efficacy of standard slit-lamp shield versus a new shield designed for increased clinician comfort as well as adequate protection.

**Methods:**

Simulations were performed using the commercial software Star-CCM+. Respiratory droplets were assumed to be 100% water in volume fraction with particle diameter distribution represented by a geometric mean of 74.4 (±1.5 standard deviation) µm over a 4-min duration. The total mass of respiratory droplets expelled from patients’ mouths and droplet accumulation on the manikin were measured under the following three conditions: with no slit-lamp shield, using the standard slit-lamp shield, and using our new proposed shield.

**Results:**

The total accumulated water droplet mass (kilogram) and percentage of expelled mass accumulated on the shield under the three aforementioned conditions were as follows: 5.84e-10 kg (28% of the total weight of particle emitted that settled on the manikin), 9.14e-13 kg (0.045%), and 3.19e-13 (0.015%), respectively. The standard shield could shield off 99.83% of the particles that would otherwise be deposited on the manikin, which is comparable to 99.95% for the proposed design. Conclusion: Slit-lamp shields are effective infection control tools against respiratory droplets. The proposed shield showed comparable effectiveness compared with conventional slit-lamp shields, but with potentially enhanced ergonomics for ophthalmologists during slit-lamp examinations.

## Abstract (pt)

**Introdução:**

Os oftalmologistas têm alto risco de contrair a doença do Coronavírus-19 devido à proximidade com os pacientes durante os exames com lâmpada de fenda. Usamos um modelo de computação para avaliar a eficácia das proteções para lâmpadas de fenda e propusemos uma nova proteção ergonomicamente projetada.

**Métodos:**

As simulações foram realizadas no software comercial Star-CCM +. Os aerossóis de gotículas foram considerados 100% de água em fração de volume com distribuição de diâmetro de partícula representada por uma média geométrica de 74,4 ± 1,5 (desvio padrão) µm ao longo de uma duração de quatro minutos. A massa total de gotículas de água acumulada no manequim e a massa expelida pela boca do paciente foram medidas em três condições diferentes: 1) Sem protetor de lâmpada de fenda, 2) com protetor padrão, 3) Com o novo protetor proposto.

**Resultados:**

A massa total acumulada das gotas de água (kg) e a porcentagem da massa expelida acumulada no escudo para cada uma das respectivas condições foram; 1) 5,84e-10 kg (28% do peso total da partícula emitida que assentou no manequim), 2) 9,14e-13 kg (0,045%), 3,19e-13 (0,015%). O escudo padrão foi capaz de proteger 99,83% das partículas que, de outra forma, teriam se depositado no manequim, o que é semelhante a 99,95% para o projeto proposto.

**Conclusão:**

Protetores com lâmpada de fenda são ferramentas eficazes de controle de infecção contra gotículas respiratórias. O protetor proposto mostrou eficácia comparável em comparação com os protetores de lâmpada de fenda convencionais, mas potencialmente oferece uma melhor ergonomia para oftalmologistas durante o exame de lâmpada de fenda.

## INTRODUCTION

The coronavirus disease 2019 (COVID-19)^(1)^ is primarily spread via respiratory droplets upon close contact with infected individuals. Studies have revealed that COVID-19 could spread through physical contact between contaminated surfaces and mucosal membranes such as eyes and mouth^(2,3)^.

Healthcare professionals are at a higher risk of severe acute respiratory syndrome coronavirus 2 infection^(4)^. An ophthalmic assessment can involve examinations such as biomicroscopy and fundoscopy, which use the slit-lamp^(5)^. The aforementioned procedures are likely to increase the risk of virus transmission given the proximity between ophthalmologists and patients in these interactions^(6,7)^. The American Academy of Ophthalmology has advised ophthalmologists to wear face masks during slit-lamp examinations^(8,9)^.

There has been rapid, widespread deployment of slit-lamp shields to reduce droplet transmission between ophthalmologists and patients. However, limited evidence is available regarding the efficacy of these slit-lamp shields. Owing to possible violations in ethical considerations while conducting efficacy trials of slit-lamp shields involving real patients and doctors, alternative methods have been used to simulate real-life scenarios^(10-13)^. Some studies have used spray cans to simulate the movement of respiratory droplets from a hypothetical patient. A major limitation of this approach is the limited evidence base to suggest that particle movement from the spray can is truly representative of respiratory droplet movement from humans^(10-13)^. The aim of our study is to evaluate the efficacy of the standard slit-lamp shield (A) in comparison to a proposed novel slit-lamp shield designed for greater clinician comfort (B) in preventing the transmission of respiratory droplets. For this purpose, we used computational models to recreate the movement of respiratory droplets.

## METHODS

A theoretical-experimental study was conducted to determine the performance of a standard slit-lamp shield and a new slit-lamp shield for optimizing the safety and ergonomics for ophthalmologists. Our design was based on the BQ 900 Slit-Lamp, Haag-Streit Holding AG, Köniz, Switzerland (312 × 305 × 676 mm). We also used the dimensions for the Extended Breath Protecting Shield, BQ 900/ BP 900 - Print on A3 available on https://hsuk.co/breathshieldtemplate. 

To evaluate the effectiveness of the slit-lamp shield in shielding respiratory droplets, 3D Computational Fluid Dynamics simulations were performed using the commercial software Star-CCM+. The conservation laws for a continuum can be expressed using a Eulerian or a Lagrangian approach. In the Eulerian approach, a given volume represents a portion of space through which material can flow. In contrast, in the Lagrangian approach, a given volume represents a portion of the material in the body, so that the observer follows the material as it moves through space.

The simulation framework adopted in this work is based on a Lagrangian-Eulerian approach where the conservation equations of mass and momentum for the dispersed phase (respiratory droplets) are written for each individual particle in Lagrangian form. This approach allowed us to calculate the trajectory of each individual particle. The equations for determining the continuous phase (air) are expressed in the Eulerian form. A two-way coupling technique is used to account for the effects of the dispersed phase on the continuous phase.

The k-epsilon turbulence model has been used to provide closure relations to the Reynolds-averaged Navier-Stokes equations, whose general form can be written as shown below:

$$\frac{\partial u}{\partial t}+u\cdot \nabla u=g-\frac{1}{\rho }\nabla p+v\nabla^{2}u$$

where **u** is the velocity vectorcosity and, **g** is the acceleration vector due to a body force, **p** is pressure, ν is the kinematic, and vρ is the density.

The change in the momentum of a particle is balanced by surface and body forces acting on it. Therefore, the conservation equation of momentum can be denoted using the following equation:

$$m_{p}\frac{dv_{p}}{dt}=F_{s}+F_{b}$$

Where **m**_p_ denotes the mass of the particle, **V**_p_ is the instantaneous particle velocity, **F**_s_ is the resultant of the forces acting on particle surface (drag force and pressure gradient force), and **F**_b_ is the resultant of body forces (gravity force, contact force, and Coulomb force).

The computational domain for the base model is represented by a 2 × 2 × 2 m box and a digital form of human head positioned at the average seated height of a human. Box dimensions were selected to avoid no-slip walls effect. The head model in this study complied with the technical specification standard ISO 16976-2^(14)^ and was based on the digital model of a medium-sized American head obtained from the National Institute for Occupational Safety and Health database^(15)^.

Expiration flow and respiratory droplets were added to the domain through a round surface of 2 cm^2^ on the wall, representing patient mouth facing directly the head model^(16)^. This injector surface was positioned at the same height as the model’s mouth and was 30 cm apart, which simulated the average distance from doctor to patient in a regular slit-lamp setup.

The shielded model face opposite to the injector face was open to the atmosphere. No ventilation air was supplied to simulate a quasi-quiescent environment, such as that used by Li et al.^(17)^. The domain was discretized using polyhedral cells with refined mesh near the injection area and the head model boundaries. Owing to the symmetrical nature of the airflow field only half the domain was required for computation.

The initial velocity profile of the air expelled through the mouth was derived from Zhang et al.^(18)^. For our study, respiratory droplets were assumed to be 100% water in volume fraction, with particle diameter distribution represented by a lognormal function with a geometric mean of 74.4 µm and a standard deviation of 1.5, based on the study by Han et al.^(19)^. A total amount of 2.08e-9 Kg of respiratory droplets was injected into the domain.

The boundaries were assumed to be adiabatic, and the expelled airflow temperature was 35°C^(18)^. Evaporation was not modeled in this study; therefore, droplet diameters did not change along the course of the simulation. The discrete phase was set to stick to the boundaries for allowing computation of the mass impingement on the doctor’s face and on the shield.

For our proposed new shield, we attempted to determine the most favorable design and maintain maximum ergonomics, based on the average dimensions of the human head^(14)^. The shield’s shape was designed to avoid air vortexing at its edges and the descent of particles owing to gravity on the doctor’s head, based on the biophysical properties of the dispersion of droplets from human cough and sneeze in published literature^(18)^. The software used for modeling the shield was Autodesk Fusion 360.

**Figure 1:**
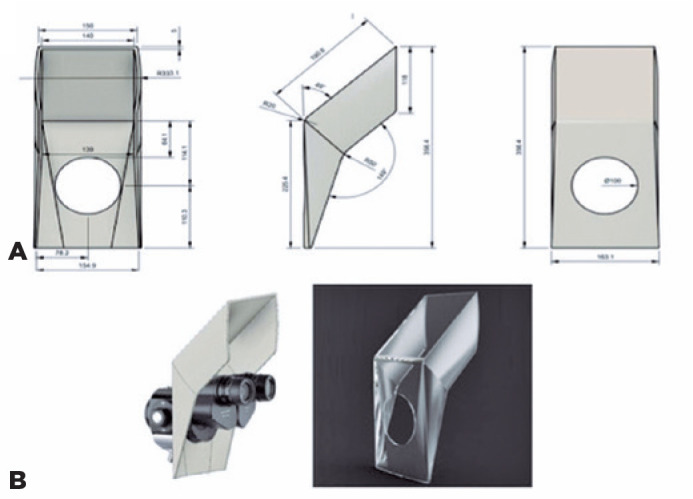
Illustration of the measurements of the proposed shield.

The recommended design uses an “on purpose” deformation on the shield edge to alter flow direction by the edge angles. Deliberate deformation on shield edge was used to reduce the number of particles that flow toward the “inside” of the shield, due to turbulent flow. The altered flow direction diverts the air and water droplets to the “outside” of the shield. This prevents direct flow and water droplets from flowing directly onto the doctor’s face. The shield dimensions were designed based on the measurements of an average human head. Using both male and female head shapes, it was possible to get an average total measurement of parameters such as the distances between the eyes, head width, and head length. With the average parameters of each dimension, which included the front and side of the head shape, a final dimension of 15 × 36 cm (width × height) was used for the shield. Additionally, the shield has a 49° bend that would extend just above the clinician’s forehead, as shown in figure 1A.

(A) Illustration of the measurements of the proposed shield. (B) Left image illustrates how the proposed shield will fit around the slit-lamp. The right image displays a 3D image of the proposed shield. These are the original figures drawn by the author; therefore, permission is granted for publishing and reproducing this figure.

The positioning of the device in the slit-lamp was based on the distance between the main controls and the slit-lamp adjustment mechanisms. We propose that the new shield be positioned between the eyepiece and objective lens of the slit-lamp. The shield was placed as close as possible to the patient’s face to maximize the barrier effectiveness. Moreover, the new positioning provides greater comfort to the clinician. In contrast, the standard shields in current practice are positioned at the oculars and closer to the ophthalmologist, which is not only less effective but also compromises comfort (Figure 1B).

For a standard slit-lamp examination, three simulations were considered: (1) without protection, (2) with the standard shield, and (3) with the proposed shield. The simulation case for the standard shield was built by adding the shield geometry to the base model, 3 cm away from the manikin’s nose, in the x-direction, for mimicking the installation in the binocular’s region. For the proposed shield, the geometry was positioned 10 cm away from the manikin’s nose. The mass of respiratory droplets accumulating on doctor’s faces for 240 s (4 min) of the simulation was chosen as the objective metric for assessing shielding effectiveness. This calculation time was representative of the average time doctors spend seated in the slit-lamp in front of the patient in a typical consultation. The accumulated mass on the shields was also monitored in respective simulated cases.

## RESULTS

### Without protection at the slit-lamp

**Figure 2:**
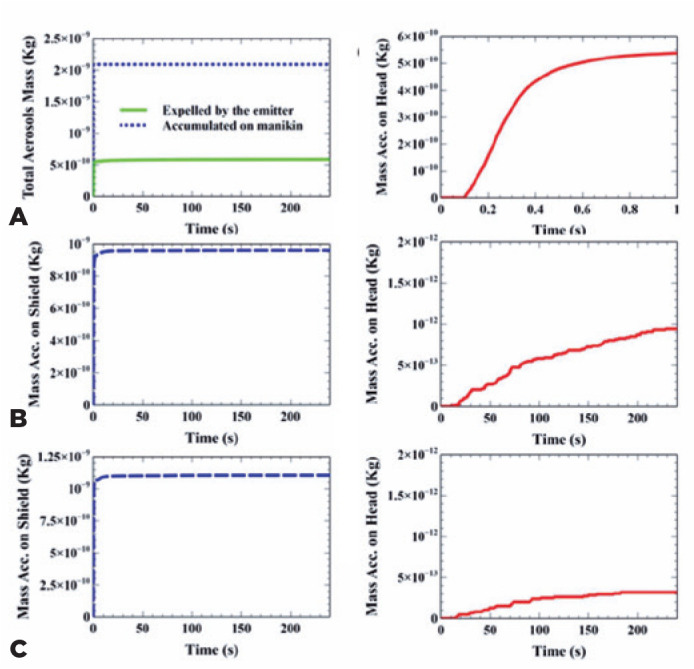
Expelled and accumulated mass in the computer simulation of the aerosol spread over the face shield and the user’s head.

The total mass of respiratory droplets accumulated on manikin surfaces and the mass expelled through patient mouths were plotted as a function of time (Figure 2), with no shield at the slit-lamp between patient and doctor. The plot clearly showed that without protection, approximately 28% of the mass expelled from respiratory droplets reached and adhered to the doctor’s head.

The total droplet mass expelled by the emitter and accumulated on the head when no protection was used throughout the whole simulation (a) and mass accumulated on the head during the first 1 s of solution (b). (c) And (d) show the total droplet masses accumulated on shield (a) and manikin (b) surfaces, respectively, throughout the simulation case with the standard shield. Total aerosols masses accumulated on shield (e) and manikin surfaces throughout the simulation case with the proposed shield (f). Original figures with permission from Edinilson Costa.

**Figure 3:**
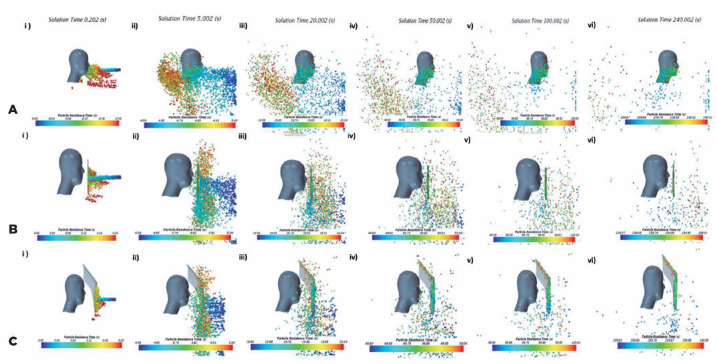
Droplet distribution in the computer simulation of aerosol spread over the face shield and the user’s head.

Particle distribution was colored based on residence time, as depicted in Figure 3A. Residence time corresponds to the exact time from when the particles are released from the emitting source. At 0.20 s, a “cloud” of particles form due to the increased airflow velocity when colliding with the manikin surface. At 5 s, particles with sizes >30 µm begin to descend, whereas smaller particles advance toward and around the head. At 200 s and 240 s, when the environment is more or less quiescent, small particles with an approximate diameter of 1 µm could remain suspended in the air for longer. However, deeper analysis of this extended behavior is beyond the scope of this study. Simulation duration to assess slit-lamp shield effectiveness was chosen as the representative of the length of a typical ophthalmic consultation.

(A) Aerosol droplet distribution colored by residence time for simulations with no shield protection, at solution times of (i) 0.20 s, (ii) 5 s, (iii) 20 s, (iv) 50 s, (v) 100 s, and (vi) 240 s. (B) Aerosol droplet distribution colored by residence time for simulations with standard shield protection, at solution times of (i) 0.20 s, (ii) 5 s, (iii) 20 s, (iv) 50 s, (v) 100 s, and (vi) 240 s. (C) Aerosol droplet distribution colored by residence time for simulation with proposed shield protection, at solution times of (i) 0.20 s, (ii) 5 s, (iii) 20 s, (iv) 50 s, (v) 100 s, and (vi) 240 s.

These are original figures drawn by the author; therefore, permission is granted for publishing and reproducing this figure.

### Using the standard shield

The total mass of respiratory droplets accumulating on manikin surfaces and on the shield, when using the standard shield, is presented as a function of the time in Figure 2. The plot shows that 9.6e-10 Kg of respiratory droplets were deposited on the shield (45.9% of mass expelled from aerosols), while 9.14e-13 Kg adhered to the manikin head (0.045% of mass expelled from aerosols).

Particle behavior and distribution analysis colored by residence time are depicted in Figure 3B. At 0.20 s, particles hit the shield surface, while particles >100µ strayed down from the main jet. At 5 s, big particles continued to descend, while particles <20 µ started to climb up the shield. At 20 s and 50 s, the animation showed how, from then on, the particles continued to move over and around the shield; some of these reached the surface of the manikin. Again, at 240 s, there were quite a large number of small droplets suspended in the air. The same environmental considerations, including particle sizes and simulation time made for the previous simulation case, were also applied here.

### With the proposed shield

The total mass of respiratory droplets accumulating on manikin surfaces and on the proposed shield as a function of the time is presented in figure 2. We found that 1.11e-9 Kg of respiratory droplets deposited on shield (53% of mass expelled from aerosols), while 3.19e-13 Kg of droplets adhered to the manikin’s head (0.015% of mass expelled from aerosols).

Particle distribution colored by residence time is depicted in Figure 3C. At 0.20 s, a stream of particles smashed into the shield surface while particles >100 µ strayed down from the main jet. At 5 s, big particles descended, while particles <20 µ move up and sideways of the shield. From 20 s to 240 s, particles continued to move over and around the shield, resulting in some reaching the surface of the manikin.

The simulation was performed using a deterministic instrument without any stochastic variable. Therefore, it was neither necessary nor possible to conduct further statistical analysis. The compilation of the total accumulated mass on the manikin in all simulation cases, expressed in kilograms, and in terms of the percentage of mass ejected through the emitter mouth, is shown in table 1.

**Table 1:** Total accumulated mass on the manikin in all simulation cases and the percentage of mass ejected through the emitter’s mouth

Case	Mass accumulated on the manikin	
	Total accumulated mass (kg)	Percentage of mass expelled by the emitter
Without protection	5·85e-10	28%
Standard Shield	9·14e-13	0·045%
Proposed Shield	3·19e-13	0·015%

## DISCUSSION

We used computational modeling to simulate the behavior of respiratory droplets during a slit-lamp examination of two slit-lamp shields. Having used published data of respiratory droplet behavior, we ensured both a safe and evidence-based scientific analysis regarding the movement of respiratory droplets and its interactions with slit-lamp shields. We demonstrate that in the absence of a shield, an ophthalmologist is at a high risk of exposure to respiratory droplets. In the present study, the mass of particles that reached the mannequin head was 5.85 e-10 kg. With the presence of the standard slit-lamp shield, the total mass of particles that reached the manikin head was 9.14e-13 kg; this is only 0.16% of what would have accumulated on the mannequin head without any shield. These findings are in line with multiple other publications that have investigated slit-lamp shields^(10-13)^. A significant strength of our study compared with other studies, such as that by Liu et al. who have also demonstrated the protective utility of slit-lamp shields, is that the simulations we used to mimic respiratory droplet movement and transmission was evidence-based using computational modeling^(13)^.

There are several models of slit-lamp shields available in the market. They are generally designed on a sheet of A3 paper. However, a significant limitation of most of these models was ophthalmologist dissatisfaction, as they interfered with arm/hand movements during slit-lamp examinations. In light of this, our aim was to develop a slit-lamp shield with maximum efficacy in alleviating the risk of COVID-19 transmission to the clinicians while simultaneously meeting satisfactory ergonomics. To achieve this, we considered the average head diameter to obtain the model proposed in this study. Shield design was a crucial component in our study. We used the minimum size deemed safe^(20)^ and designed it according to the airflow and behavior of respiratory particles. The final shape of our new proposed shield was curved to facilitate a vortex effect to minimize continuous flow of air for mitigating the transmission of respiratory droplets. The shield’s position was chosen according to a location that would confer maximum protection to the ophthalmologist at the closest possible distance to the patient. The rationale behind this was to minimize the dispersion of respiratory droplets with minimal compromise to the clinician’s comfort and dexterity while using the slit-lamp. Considering the number of respiratory droplets accumulated on the manikin without any slit-lamp shield as 100%, the standard shield could prevent 99.83% of the respiratory droplets that would have otherwise been deposited on the manikin. Moreover, the proposed new shield design was successful in preventing the deposition of 99.95% of respiratory droplets on the manikin. So, the proposed shield could shield a further 30% of the particles juxtaposed to the standard shield. The results demonstrate that although the mass of the contamination was relatively small, neither of the evaluated shields was capable of preventing 100% of respiratory droplets from reaching the manikin. The simulation was performed using a deterministic instrument without any stochastic variable. Therefore, it was neither necessary nor possible to conduct further statistical analysis.

There were some limitations to the study. The evaluation of COVID-19 transmission through droplets that was conducted was based on large respiratory particles. The number of droplets that fell onto other body parts has not been accounted for in this study, and more simulations are required to investigate this. Moreover, although the proposed shield design was based on experiences of ophthalmic experts, it was not formally assessed for its ergonomic superiority. Further work is required to formally investigate the ergonomic benefit it confers. Furthermore, the shape of the slit-lamp was not considered in the computerized simulation as there are several commercial models available. The economic implications of the proposed new shield have not been evaluated; however, given that it is smaller than the standard shield, we expect it to be more cost effective given that it would require less raw materials for production. To conclude, this study used computational modeling to simulate the natural movement of respiratory particles to demonstrate the efficacy of slit-lamp shields in preventing the transmission of respiratory droplets during ophthalmic examination. While the standard slit-lamp shield has demonstrated efficacy in protection, it compromises clinicians’ comfort and manual dexterity. The proposed new shield was designed based on the opinion of ophthalmic experts to afford greater comfort to clinicians and facilitate their manual handling. Moreover, safety tests have determined the proposed new shield to be more effective in preventing transmission of respiratory droplets. Looking to the future, slit-lamp shields will not only be useful in mitigating the risks of COVID-19 transmission but also other severe acute respiratory syndromes. The COVID-19 pandemic will be an impetus in promoting global adoption of protection of this nature.
